# Outcomes Following Colorectal Cancer Resection in Elderly Patients

**DOI:** 10.3390/curroncol32120652

**Published:** 2025-11-21

**Authors:** Richard Grainger, Tatiana S. Temperley, Hugo C. Temperley, Ben Creavin, Emily Harrold, Cillian Clancy, James O’Riordan, David Gallagher, Brian J. Mehigan, John Larkin, Charles Gillham, Dara Kavanagh, Paul H. McCormick, Michael E. Kelly

**Affiliations:** 1 Department of Surgery-Trinity college Dublin/St James Hospital Dublin Ireland D08 NHY1 Dublin, Ireland; 2 Department of Radiology, St. James’s Hospital, D08 NHY1 Dublin, Ireland; 3 Trinity St. James’s Cancer Institute, D08 NHY1 Dublin, Ireland; 4 Department of Medical Oncology, St. James’s Hospital, D08 NHY1 Dublin, Ireland; 5 Department of Genetics, St. James’s Hospital, D08 NHY1 Dublin, Ireland; 6 Department of Radiation Oncology, St. James’s Hospital, D08 NHY1 Dublin, Ireland

**Keywords:** colorectal cancer, elderly, surgical outcomes, comorbidity, postoperative complications, frailty

## Abstract

**Background:** Colorectal cancer (CRC) mainly affects older adults, yet elderly patients are underrepresented in outcomes research. Accurate risk stratification tools, such as the Charlson Comorbidity Index (CCI), are essential for guiding surgical decisions in this group. **Methods:** We conducted a retrospective review of patients aged 75 years or older who underwent colorectal cancer resection at a tertiary centre between January 2019 and September 2024. Clinical, pathological, and molecular data were analyzed. The primary outcome was a composite of major postoperative complications (Clavien–Dindo grade 3 or higher) or 30-day mortality, stratified by CCI (5 or higher vs. less than 5). Statistical tests included chi-square, Fisher’s exact, and Mann–Whitney U as appropriate. **Results:** The median age was 81 years (range 75–97), with 59.7% male. CCI ≥ 5 was observed in 24.6% (51/211). The primary composite outcome of major postoperative complications or 30-day mortality occurred in 15/51 (29.4%) patients with a CCI ≥ 5 compared to 19/160 (11.9%) with a CCI < 5 (*p* = 0.04). Major complications occurred in 18.5% (39/211) of cases, and the 30-day mortality rate was 3.3% (7/211). Laparoscopic resection was independently protective in multivariate analysis (adjusted OR 0.37, *p* = 0.048), while age ≥85 and emergency presentation were not statistically significant predictors. **Conclusions:** Colorectal resection in patients aged ≥75 is linked with acceptable morbidity and low short-term death rates. A CCI ≥ 5 significantly predicts adverse outcomes and should be included in preoperative assessments. Minimally invasive surgery seems advantageous and should be considered, when possible, to enhance results in this high-risk group.

## Simple Summary

Colorectal cancer is a common disease in older adults, but older patients are often not included in research studies. As a result, we do not always know how well they recover after surgery or which factors predict a difficult recovery. In this study, we looked at over 200 patients aged 75 and older who had surgery for colorectal cancer at a large hospital in Ireland. We collected information about their health before surgery, the type of operation they had, and how they did afterwards. We found that surgery was generally safe, even for older patients. However, people with more medical conditions were more likely to have serious problems after surgery or to die within 30 days. We also found that patients who had laparoscopic (keyhole) surgery tended to recover better. Our findings show that age alone should not stop someone from having potentially life-saving surgery. Instead, doctors should consider the patient’s overall health. This information can help doctors and patients make better decisions together and may lead to more older adults being offered treatment that can improve their survival and quality of life.

## 1. Introduction

CRC remains a significant global health issue, ranking among the top causes of cancer-related illness and death, with over 1.9 million new cases and nearly one million fatalities annually. The incidence of CRC is strongly associated with ageing, and demographic trends indicate that most new diagnoses occur in individuals over 70, with a sharp increase in cases among those aged 80 and older as populations continue to age worldwide [1,2,3]. This epidemiological shift is expected to significantly increase the burden of CRC in elderly patients over the coming decades, presenting major challenges for healthcare systems and clinical practice management [1,2,3].

Surgical resection remains the primary treatment for localized CRC, and strong evidence indicates that elderly patients, including those aged 80 years or older, can achieve meaningful survival benefits from surgery when carefully chosen [4,5,6]. However, elderly patients are often regarded as high-risk surgical candidates because they tend to have more health problems, reduced physiological capacity, greater frailty, and a higher likelihood of postoperative complications such as pneumonia, delirium, and extended hospital stays [1,3,5,7,8,9]. These concerns often result in therapeutic nihilism or overly cautious management, despite growing evidence suggesting that age alone should not be a contraindication for surgery [3,5,6,8]. Notably, long-term survival after recovering from the initial postoperative period is similar to that of younger patients, emphasizing the importance of careful perioperative management and patient selection [6,9].

The literature on CRC surgery in the elderly is limited because this group is underrepresented in clinical trials and there is inconsistent use of comprehensive comorbidity indices, frailty assessments, and molecular tumour characteristics, all of which can affect prognosis and treatment response [1,2,10,11]. Recent studies emphasize the importance of personalized risk assessment using validated tools, such as the American Society of Anaesthesiologists (ASA) score and the Charlson Comorbidity Index. They also emphasize the importance of multidisciplinary evaluation, including geriatric liaison and comprehensive geriatric assessment, to enhance outcomes and support informed decision-making [1,3,5,7,11]. Furthermore, there is increasing recognition of the importance of person-centred outcomes, such as postoperative quality of life, maintaining independence, and discharge destination, which are especially relevant for the elderly population but remain underreported in the literature [11].

Despite an expanding body of literature on colorectal surgery in older adults, relatively few investigations have combined comorbidity, frailty, and tumour-related molecular features into a unified perioperative risk profile. Recent work shows that frailty independent of chronological age, is strongly associated with elevated postoperative mortality and morbidity in older patients undergoing colorectal cancer surgery and that frailty assessed via a modified frailty index correlates with worse short-term surgical outcomes [12,13]. Emerging evidence also highlights the benefit of geriatric co-management and comprehensive assessment in improving postoperative outcomes in older surgical oncology populations [14,15]. The present study builds on this foundation by integrating comorbidity burden, operative factors, and mismatch repair (MMR) tumour status to examine their combined influence on short-term outcomes, aligning with the evolving practice of “precision surgery” where host physiology, frailty profile, and tumour biology are assessed together to guide decision-making in older adults.

As surgical outcomes improve, attention has shifted toward identifying which older adults benefit most from curative resection versus alternative management. Exploring how comorbidity relates to tumour biology may provide additional context for risk assessment and patient counselling, particularly as health systems adapt to caring for an ageing surgical population.

As the population ages, continuous research is vital to refine risk stratification, enhance perioperative care, and ensure equitable access to evidence-based surgical treatment for elderly CRC patients. This study aims to address key gaps by examining a clearly defined group of elderly CRC patients, integrating clinical, pathological, and molecular data to evaluate short-term outcomes and identify predictors of poor prognosis, thereby guiding future strategies for personalized care and improved patient-centred results [2,7,9,16].

## 2. Methods

### 2.1. Study Design and Population

This was a retrospective observational study conducted at a single tertiary hospital. Patients aged 75 years or older who underwent elective or emergency surgical resection for histologically confirmed colorectal cancer (mainly adenocarcinoma, including rare neoplasms such as carcinoid/neuroendocrine tumours) between January 2019 and September 2024 were eligible for inclusion.

All data were de-identified and anonymized before analysis. Ethical approval for the study was obtained from the SJH/TUH Joint Research Ethics Committee (Registration No. 122).

### 2.2. Inclusion Criteria

- Age ≥ 75 years at time of surgery.
- Histologically confirmed colorectal cancer.
- Underwent curative-intent resection (elective or emergency).

### 2.3. Exclusion Criteria

- Palliative procedures without tumour resection.
- Incomplete pathological or operative data (minimal missing data tolerated if outcome data were present).

### 2.4. Data Collection

Data were extracted from electronic health records, pathology reports, radiology systems, and operative notes. Variables included demographics (age, sex), comorbidities (Charlson Comorbidity Index [CCI], ASA classification), operative details (approach, urgency, procedure type), tumour features (site, histology, pTNM staging, lymphovascular invasion [LVI], perineural invasion [PNI], margin status), molecular data (MMR, KRAS, NRAS, BRAF status) and postoperative outcomes (Clavien–Dindo grade, 30-day mortality and length of stay).

### 2.5. Outcome Measures

The primary composite outcome was the incidence of major postoperative complications (Clavien–Dindo grade ≥ 3) or 30-day mortality, stratified by Charlson Comorbidity Index (CCI ≥ 5 vs. <5), to assess the impact of comorbidity burden on surgical outcomes in elderly CRC patients. Major complications included those requiring surgical, endoscopic, or radiological intervention (Clavien–Dindo grade 3a or 3b) or life-threatening events necessitating intensive care unit management (grade 4).

### 2.6. Statistical Analysis

Descriptive statistics were presented as medians with interquartile ranges (IQRs) for continuous variables and frequencies with percentages for categorical variables. The primary outcome was analyzed using chi-square or Fisher’s exact test to compare rates of major complications or 30-day mortality between CCI ≥ 5 and CCI < 5 groups. Other comparisons (e.g., age ≥ 85 vs. <85 years) used chi-square, Fisher’s exact, or Mann–Whitney U tests as appropriate.

Missing data were managed using complete-case analysis, as the overall proportion of missing values was low. Major postoperative complications were defined according to the Clavien–Dindo classification, with grade III or higher events considered clinically significant. The Charlson Comorbidity Index (CCI) was analyzed both as a continuous variable and as a categorical variable, with a threshold of ≥5 used to indicate high comorbidity burden. This cut-off corresponded approximately to the upper quartile of our cohort distribution and provided clear clinical stratification. Logistic regression diagnostics were performed to confirm model stability and calibration, with all analyses performed using standard statistical software and validated methods.

## 3. Results

### 3.1. Patient Characteristics and Comorbidities

A total of 211 patients aged 75 years or older were included. The median age was 81 years (range 75–97). Eighty-five patients (40.3%) were aged 75–80 years, 93 (44.1%) were over 80 years, and 50 (23.7%) were 85 years or older. Overall, 59.7% of the participants were male (126/211). The median Charlson Comorbidity Index (CCI) was 4 (IQR, 3–5), with 74.9% (158/211) scoring four or higher. ASA classification was II in 83.6% (168/201), III in 11.9% (24/201), and IV in 4.5% (9/201). Common comorbidities included chronic pulmonary disease (17.1%), diabetes mellitus (15.6%; 45.5% with end-organ damage), congestive heart failure (9.0%), prior myocardial infarction (6.0%), and peripheral vascular disease (4.5%) (Table 1).

### 3.2. Tumour and Operative Characteristics

Tumours were predominantly rectal (43.1%, 91/211), followed by right-sided (35.5%, 75/211) and left-sided (21.3%, 45/211) colon cancers. Neoadjuvant therapy was administered in 53 cases, the majority of whom had rectal cancer (n = 43), with the remainder comprising left-sided (n = 6) and right-sided (n = 4) tumours. Most resections were laparoscopic (168/211, 79.6%), including one robotically assisted, with 28/211 (13.3%) performed open and 14/211 (6.6%) recorded as local excisions. The majority were elective (182/211, 86.3%), with 29/211 (13.7%) performed as emergencies. The median postoperative length of stay was 12 days (IQR, 7–20). Adenocarcinoma not otherwise specified (NOS) was the predominant histology (91.4%, 193/211), followed by mucinous adenocarcinoma (6.6%, 14/211) and carcinoid/neuroendocrine tumours (1.9%, 4/211) (Table 2). Adjuvant therapy was administered in 36 cases, 35 of whom received chemotherapy and/or immunotherapy. One patient received combined chemoradiotherapy.

### 3.3. Pathological Findings

The most common pathological stage was pT3 (48.3%, 102/211), followed by pT2 (27.0%, 57/211), pT4 (17.5%, 37/211), and pT1 (7.1%, 15/211). Nodal involvement was present in 41.2% (87/211; pN1: 28.6% [60/211], pN2: 12.6% [27/211]). Metastatic disease (M1) was found in 7.6% (16/211), including liver (n = 3), lung (n = 3), peritoneal (n = 2), renal (n = 1), bladder (n = 1), and multiple sites (n = 3); three cases had metastatic disease recorded without specified location. Lymphovascular invasion was identified in 18.6% (39/211) (Table 3).

### 3.4. Molecular Characteristics

Among the 25 patients with MMR deficiency, BRAF status was available for three patients. All of these had a BRAF mutation, suggesting a likely sporadic origin in these cases. However, BRAF testing was incomplete (available in only 12% of MMR-deficient patients), limiting definitive conclusions (Table 4).

### 3.5. Postoperative Outcomes

The primary composite outcome of major postoperative complications (Clavien–Dindo grade ≥ 3) or 30-day mortality, stratified by CCI, occurred in 29.4% (15/51) of patients with CCI ≥ 5 compared to 11.9% (19/160) with CCI < 5 (*p* = 0.04). Overall, major complications occurred in 18.5% (39/211), including surgical site infections, sepsis, cardiorespiratory deterioration, and anastomotic leaks (n = 4). Thirty-day mortality was 2.8% (6/211), with recorded causes including perforated gastric ulcer (n = 1), liver recurrence (n = 1), and COVID-19 infection (n = 2). The significant association between CCI ≥ 5 and the primary outcome highlight the critical role of comorbidity burden in predicting adverse postoperative events (Table 5).

### 3.6. Subgroup Analyses

Beyond the comorbidity burden (CCI ≥ 5), which was significantly associated with adverse outcomes (Table 4), further subgroup analyses were performed. Subgroup analyses of the primary endpoint (major complications ≥ 3 or 30-day mortality, Table 5) demonstrated that emergency surgery was associated with a higher event rate than elective surgery (38.9% vs. 20.6%; OR 2.47, 95% CI 0.89–6.87, *p* = 0.084). Laparoscopic resections had significantly fewer events than open resections (19.4% vs. 43.5%; OR 0.31, 95% CI 0.12–0.79, *p* = 0.014). Age stratification (75–80, 81–85, ≥86 years) showed composite outcome rates of 8.2% (8/98), 8.2% (5/61), and 11.5% (6/52), respectively, and right- vs. left-sided resections (OR 0.56, 95% CI 0.20–1.55, *p* = 0.262) were not significant. In multivariate analysis, including emergency status, age, tumour laterality, and operative approach, laparoscopic resection remained independently protective (adjusted OR 0.37, 95% CI 0.14–0.99, *p* = 0.048) (Table 6)

When major complications were analyzed separately (Table 7), the findings were consistent. Laparoscopic resection was associated with fewer complications compared to open (OR 0.29, 95% CI 0.12–0.75, *p* = 0.010), while emergency procedures again showed a trend toward worse outcomes (OR 2.37, 95% CI 0.85–6.59, *p* = 0.099). Age stratification showed major complication rates of 8.2% (8/98), 4.9% (3/61), and 9.6% (5/52) across 75–80, 81–85, and ≥86 years, respectively.

### 3.7. Elderly-Specific Risk Model

To enhance the predictive utility of the Charlson Comorbidity Index (CCI) beyond its standalone application, we developed a simple, interaction-based risk stratification model tailored to elderly colorectal cancer patients by combining CCI ≥ 5 and emergency surgical presentation, the two strongest predictors in our cohort. Patients were categorized into three clinically intuitive risk tiers (Table 8).

− Low risk: CCI < 5 and elective surgery (n = 143, 67.8%): composite outcome (major complication ≥ 3 or 30-day mortality) in 10.5% (15/143).
− Intermediate risk: either CCI ≥ 5 with elective surgery or CCI < 5 with emergency surgery (n = 56, 26.5%): event rate of 26.8% (15/56; OR 3.12, 95% CI 1.40–6.95, *p* = 0.005 vs. low).
− High risk: CCI ≥ 5 and emergency surgery (n = 12, 5.7%): event rate of 58.3% (7/12; OR 12.0, 95% CI 3.42–42.1, *p* < 0.001 vs. low).

The model demonstrated excellent discrimination, with a χ^2^ for trend of 26.13 (*p* < 0.001), confirming a clear monotonic increase in risk across tiers. While emergency cases (n = 29, 13.7%) represent life-saving interventions where surgical decision-making is non-discretionary, the primary clinical value of this model lies in the elective setting (86.3%), where CCI ≥ 5 identifies a high-risk subgroup (n = 39) with a 2.6-fold increase in adverse events (26.8% vs. 10.5%), offering a critical window for preoperative optimization, geriatric assessment, enhanced recovery protocols, or shared decision-making.

### 3.8. Integration of Molecular Data: MMR Status and CCI Interaction

MMR status was available in 163 of the 211 patients (77.3%), with 25 (15.2%) showing MMR deficiency, as summarized in Table 4. We examined whether MMR status modified the effect of high comorbidity burden (CCI ≥ 5) on the composite outcome of major postoperative complication or 30-day mortality.

The interaction was statistically significant (*p* = 0.045). Among patients with low comorbidity (CCI < 5), event rates were similar regardless of MMR status: 8.0% in the 113 MMR-proficient cases and 11.1% in the 18 MMR-deficient cases. In contrast, high comorbidity markedly increased risk, but the effect was most pronounced in MMR-deficient tumours: 28.0% (7/25) in proficient cases versus 42.9% (3/7) in deficient cases (OR 8.57, 95% CI 1.67–44.0, *p* = 0.009). These findings are presented in full in Table 9.

## 4. Discussion

This study demonstrates that colorectal cancer (CRC) resection in patients aged ≥75 years is feasible and safe, with a composite primary outcome of major postoperative complications (Clavien–Dindo grade ≥ 3) or 30-day mortality occurring in 29.4% of patients with CCI ≥ 5 compared to 12% with CCI < 5 (*p* = 0.04). The 30-day mortality rate of 2.8% (6/211) is low, and the rate of major complications (18.4%, 39/211) is acceptable, supporting the safety of surgery in elderly patients when performed with careful risk stratification.

The integration of molecular phenotype further refines risk stratification in this elderly cohort. MMR deficiency, present in 15.3% of tested cases (Table 4), interacted significantly with high comorbidity burden (CCI ≥ 5), resulting in a 42.9% event rate (OR 8.57, 95% CI 1.67–44.0, *p* = 0.009; Table 8), nearly double the 28.0% risk observed in MMR-proficient patients with CCI ≥ 5. This clinically meaningful synergy suggests that immune dysregulation in MMR-deficient tumours may compound physiological frailty, amplifying surgical stress intolerance in older adults [17,18]. While MSI-high status confers prognostic benefit in early-stage, untreated colorectal cancer due to enhanced immunogenicity, elderly surgical patients appear to experience a paradoxical increase in perioperative risk, particularly when comorbid, a pattern increasingly recognized in geriatric oncology [19].

Using CCI as a stratification factor in the primary outcome improves the study’s novelty by directly addressing the need for personalized risk assessment in elderly CRC patients. This method aligns with the growing consensus that physiological reserve and comorbidity burden, rather than chronological age, should determine surgical eligibility [6,8]. Laparoscopic surgery and perioperative optimization further mitigate risks, particularly in patients with high CCI.

These findings align with international literature. Specifically, the Australia and New Zealand Binational Colorectal Cancer Audit found a 30-day mortality rate of 3.3% in patients aged ≥ 80 years, with surgical complication rates comparable to younger patients, and age not being an independent predictor of 30-day morbidity or mortality after adjustment for comorbidity [20]. When stratified into age bands (75–80, 81–85, ≥86 years), no gradient in risk emerged, reinforcing that physiological reserve, not chronological age, determines outcome. Similarly, meta-analyses confirm that although elderly patients have higher comorbidity burdens, surgery-related complications and disease-free survival are comparable to younger cohorts when risk is stratified using tools like CCI [3,5,7,9,20,21,22,23,24]. A Swedish population-based study reported increased 90-day and 1-year mortality in older patients, but no significant difference in postoperative complications or intensive care needs compared to younger patient cohorts [21]. Meta-analytic data confirm that although octogenarians have higher comorbidity and overall complication rates, surgery-related complications and disease-free survival are similar to those of younger patients, supporting individualized treatment decisions based on physiologic rather than chronological age [3].

Recent single-centre and multicentre studies further confirm that advanced age alone does not contraindicate resectional colorectal surgery, and that short-term outcomes—including major complications and mortality—are acceptable in well-selected elderly patients [5,8,22,24,25]. The decline in postoperative complications over time is mainly due to improvements in non-surgical complications, while surgical complication rates stay consistent across age groups [22]. Notably, long-term survival after recovering from the initial postoperative period aligns with that of younger patients, highlighting the importance of perioperative optimization and geriatric assessment [9,26].

In this cohort, only 17.1% of patients received adjuvant therapy, predominantly chemotherapy alone. Patients who received adjuvant treatment had slightly lower comorbidity burden (mean CCI 3.8 vs. 4.2), suggesting that patient fitness played a role in treatment decisions. However, many patients who underwent elective surgery and had modest CCI scores (≤4) did not receive adjuvant therapy. While our dataset does not include referral or MDT decision outcomes, this highlights an area for improvement in treatment equity. Given that surgical fitness often reflects adequate physiological reserve, age or comorbidity alone should not automatically preclude adjuvant therapy consideration. These findings support recent calls in the literature to reassess adjuvant decision-making in older adults with resected colorectal cancer [27].

While this study’s single-centre design ensures detailed, standardized data collection, its generalisability to other populations warrants consideration. Our postoperative outcomes are comparable to those reported in large multicentre and population-based cohorts. The Australia and New Zealand Binational Colorectal Cancer Audit documented a 30-day mortality rate of 3.3% and a major complication rate of 18% in patients aged 80 years and older, supporting the safety of colorectal resection in well-selected elderly patients [20]. Similarly, a Swedish population-based study found comparable postoperative morbidity and intensive-care use across age groups, despite higher long-term mortality in older adults [21]. Recent multicentre data from Italy further reinforce these observations: Pulvirenti et al. reported that age was not independently associated with poorer oncologic outcomes after neoadjuvant chemoradiotherapy and surgery for rectal cancer, while Marcellinaro et al. reported minimal age-related differences in “failure-to-rescue” rates following laparoscopic colorectal surgery in patients ≥75 years [28,29]. Collectively, these findings indicate that our results are consistent with international experience and likely generalizable beyond a single institution, while highlighting the value of multicentre collaboration to confirm reproducibility across diverse healthcare systems.

We acknowledge some limitations in this study, including its retrospective design, which restricts causal inferences, and the absence of long-term survival, cancer-specific outcomes, clinical frailty scores, radiological assessments of sarcopenia and nutritional data, all of which limit comprehensive risk profiling. The molecular subset (n = 150) and incomplete complication data (76/211 patients) may introduce bias. Future prospective studies should include frailty indices, nutritional status, and long-term outcomes to improve risk stratification and optimize care for this growing population.

While the single-centre nature of this analysis provides detailed, standardized data, it may also limit broader applicability. Future work could incorporate multicentre datasets or national registry linkage to confirm these findings across varied healthcare systems. Additional variables such as frailty indices, nutritional status, and social support, could further refine risk models and enhance predictive accuracy. Longitudinal follow-up would also allow exploration of how postoperative recovery and quality of life differ by comorbidity burden. These refinements would strengthen the translational potential of this work and inform perioperative guidelines for older adults with colorectal cancer.

Beyond statistical associations, these findings highlight the need for coordinated, patient-centred surgical care in older adults with colorectal cancer. Preoperative planning should incorporate comorbidity assessment, functional status, and patient priorities rather than relying solely on chronological age. Multidisciplinary input from surgery, anaesthesiology, geriatrics, and oncology can help balance oncological objectives with physiological reserve and recovery potential. Incorporating prehabilitation and nutritional optimization programs may reduce postoperative complications and support earlier return to baseline function. Broader adoption of structured perioperative assessment frameworks could also enhance consistency in risk communication and shared decision-making. Ultimately, aligning surgical interventions with individual goals of care will not only improve safety and outcomes but also promote meaningful recovery and long-term quality of life for this growing patient population.

## 5. Conclusions

Surgical resection for colorectal cancer in patients aged 75 and older shows acceptable outcomes. These findings confirm that age alone should not be a reason to prevent surgery. The Charlson Comorbidity Index is a crucial tool for preoperative risk assessment, with a CCI of 5 or higher significantly predicting poor outcomes and communicating this risk to individual patients.

## Figures and Tables

**Table 1:** Patient Characteristics and Comorbidities.

Characteristic	Value
Number of patients	211
Median age (IQR), years	81 (75–97)
Age 75–80, n (%)	85 (40.3)
Age > 80, n (%)	93 (44.1)
Age ≥ 85 (%)	50 (23.7)
Male sex, n (%)	126 (59.7)
CCI, median (IQR)	4 (3–5)
CCI ≥ 4, n (%)	158 (74.9)
ASA II, n (%)	168 (83.6)
ASA III, n (%)	24 (11.9)
ASA IV, n (%)	9 (4.5)
Chronic pulmonary disease, n (%)	36 (17.1)
Diabetes mellitus, n (%)	33 (15.6)
− with end-organ damage	15 (7.1)
Congestive heart failure, n (%)	19 (9.0)
Myocardial infarction, n (%)	13 (6.0)
Peripheral vascular disease, n (%)	10 (4.5)

CCI: Charlson Comorbidity Index; ASA: American Society of Anesthesiologists; IQR: Interquartile Range.

**Table 2:** Tumour and Operative Characteristics.

Characteristic	Value
Left-sided tumours, n (%)	45 (21.3%)
Right-sided tumours, n (%)	75 (35.5%)
Rectal tumours, n (%)	91 (43.1%)
Laparoscopic resections, n (%)	168 (79.6%)
Open resections, n (%)	28 (13.3%)
Local excisions, n (%)	14 (6.6%)
Elective procedures, n (%)	182 (86.3%)
Emergency procedures, n (%)	29 (13.7%)
Length of stay, median (IQR), days	12 (7–20)
Adenocarcinoma NOS, n (%)	193 (91.4%)
Mucinous adenocarcinoma, n (%)	14 (6.6%)
Neuroendocrine tumours, n (%)	4 (1.9%)

n (%); Number (percentage).

**Table 3:** Pathological Characteristics.

Characteristic	Value
pT1, n (%)	15 (7.1%)
pT2, n (%)	57 (27.0%)
pT3, n (%)	102 (48.3%)
pT4, n (%)	37 (17.5%)
pN0, n (%)	124 (58.8%)
pN1, n (%)	60 (28.6%)
pN2, n (%)	27 (12.6%)
Metastatic disease (M1), n (%)	16 (7.6%)
Lymphovascular invasion, n (%)	39 (18.6%)
Perineural invasion, n (%)	31 (14.7%)
R1 resection margin, n (%)	20 (9.5%)
MMR deficiency, n (%)	22 (14.9%)
KRAS mutation, n (%)	48 (31.9%)
BRAF mutation, n (%)	18 (11.8%)
NRAS mutation, n (%)	2 (1.5%)

n (%); number (percentage).

**Table 4:** Molecular Characteristics.

Molecular Markers	Prevalence
MMR Deficiency	25/163 (15.3%)
KRAS Mutation	7/20 (35%)
BRAF Mutation	3/14 (21.1%)

x/y (%), number/total available (percentage).

**Table 5:** Postoperative Outcomes.

Outcome	CCI ≥ 5 (n = 51)	CCI < 5 (n = 160)	*p*-Value
Composite outcome (major complication or 30-day mortality), n (%)	15 (29.4%)	19 (11.9%)	0.04
Major complications (Clavien–Dindo ≥ 3), n (%)	12 (23.1%)	27 (17.0%)	0.49
− Grade 3a	6 (11.5%)	13 (8.8%)	0.58
− Grade 3b	2 (3.8%)	3 (1.9%)	0.60
− Grade 4	4 (7.7%)	11 (6.9%)	0.76
30-day mortality, n (%)	3 (5.8%)	3 (1.9%)	0.170

**Table 6:** Subgroup analyses of the composite primary outcome (major complication ≥ 3 or 30-day mortality).

Subgroup	Variable	N	Major Complication ≥ 3 or 30-Day Mortality	Rate (%)	OR (95% CI)	*p*-Value
Elective vs. Emergency	Elective	160	33	20.6		
	Emergency	18	7	38.9	2.47 (0.89–6.87)	0.084
Lap vs. Open †	Lap	144	28	19.4		
	Open	23	10	43.5	0.31 (0.12–0.79)	0.014
Age	75–80	98	8	8.2		
	81–85	61	5	8.2	1.00 (0.31–3.24)	1
	≥86	52	6	11.5	1.47 (0.48–4.48)	0.5
Right vs. Left	Left	39	9	23.1		
	Right	63	9	14.3	0.56 (0.20–1.55)	0.262

† Local excisions excluded from this comparison.

**Table 7:** Subgroup analyses for major complications (Clavien–Dindo ≥ 3).

Predictor	n	Events	OR (95% CI)	*p*-Value
Emergency vs. Elective	169	39	2.37 (0.85–6.59)	0.099
Age 75–80	98	8		
Age 81–85	61	3	0.58 (0.15–2.25)	0.43
Age ≥ 86	52	5	1.19 (0.37–3.88)	0.77
Lap vs. Open †	164	38	0.29 (0.12–0.75)	0.01

† Local excisions excluded.

**Table 8:** Elderly-Specific Risk Model for Composite Outcome (Major Complication ≥ 3 or 30-Day Mortality).

Risk Group	Criteria	N	Events, n	OR (95% CI) vs. Low	*p*-Value
Low	CCI < 5 and elective	143	15		
Intermediate	(CCI ≥ 5 and elective) Or (CCI < 5 and emergency)	56	15	3.12 (1.40–6.95)	0.005
High	CCI ≥ 5 + emergency	12	7	12.0 (3.42–42.1)	<0.001
				χ^2^ for trend = 26.13, *p* < 0.001	

**Table 9:** Interaction Between CCI ≥ 5 and MMR Status on Composite Outcome.

CCI/MMR Status	N	Events, n	OR (95% CI)	*p*-Value
CCI < 5 + MMR-proficient	113	9		
CCI < 5 + MMR-deficient	18	2	1.44 (0.29–7.1)	0.66
CCI ≥ 5 + MMR-proficient	25	7	4.40 (1.55–12.5)	0.005
CCI ≥ 5 + MMR-deficient	7	3	8.57 (1.67–44)	0.0009

Interaction *p* = 0.045 (likelihood ratio test).

## Data Availability

The data presented in this study are available on reasonable request from the corresponding author. The data are not publicly available due to institutional and ethical restrictions related to patient confidentiality.
